# Supplementary material for: Analysis in vivo using a new method, ARGO (Analysis of Red Green Offset), reveals complexity and cell-type specificity in presynaptic turnover of synaptic vesicle protein Synaptogyrin/SNG-1
Source: bioRxiv. 2025 Oct 9:2024.11.26.625560. Preprint. [Version 3] doi: 10.1101/2024.11.26.625560 (PMC12632557; doi:10.1101/2024.11.26.625560)
Supplement: Supplement 1 [file media-1.pdf]

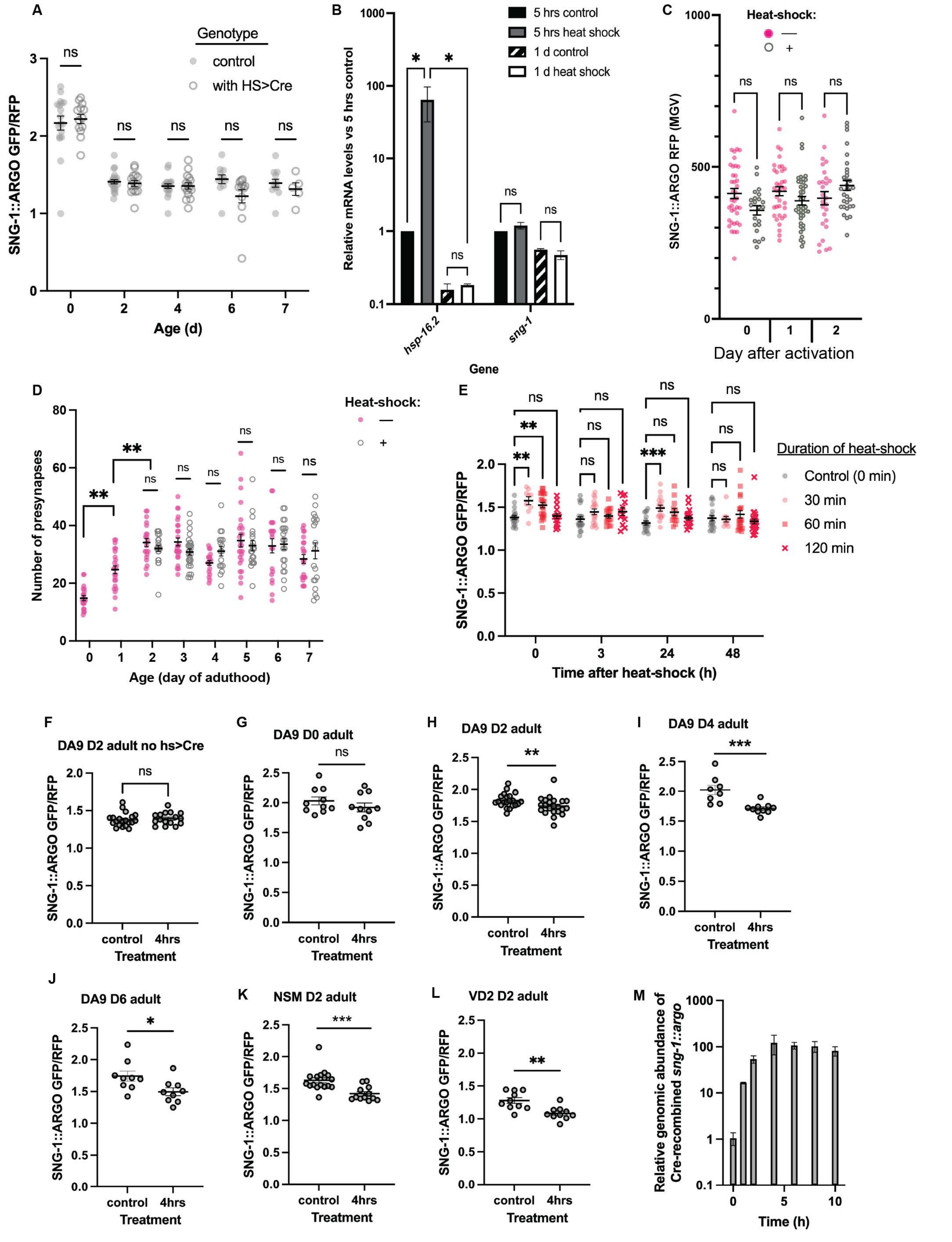

Figure S1. Validation of the heat-shock-generated pulse to express Cre recombinase. (A) Steady-state average presynaptic SNG-1::ARGO GFP/RFP in a strain without versus with the Pheat-shock>Cre transgene heSi160 indicates that there is little ectopic recombination in the absence of heat-shock. Each data point is the average of all individual presynaptic ratios from one DA9 neuron. (B) The heat-shock pulse does not discernably impact sng-1 transcript levels. qRT-PCR analysis of sng-1 mRNA levels with versus without the heat-shock conditions used as the pulse to activate SNG-1::ARGO. Values represent fold change relative to control (no heat-shock) ± SEM (n = 2 biological replicates, *P<0.05, two-way ANOVA with Tukey post-test). (C-D) The heat-shock pulse shows no significant effect on SNG-1 ARGO steady-state abundance. (C) Mean presynaptic SNG-1::ARGO RFP fluorescence intensity with versus without the heat-shock pulse. The experiment was started on A2. Each data point shows the average mean RFP intensity for all synapses of the DA9 neuron for one neuron. (D) Number of DA9 presynapses per neuron with versus without the heat shock pulse at A2. For (C-D), The heat-shocked animals at time = 0 were imaged 4 hours post heat-shock, and bars show mean ± SEM (ns (not significant): P>0.05, *P<0.5, ***P<0.001, two-way ANOVA with Tukey post-test). (E) Quantification of presynaptic SNG-1::ARGO GFP/RFP in the DA9 neuron, in a strain lacking heSi160[Pheat-shock>Cre]. Animals were heat-shocked at 34 ⊠C for the indicated time, or not at all, at adult Day 2, then periodically imaged. Each data point is average per worm; bars show mean ± SEM (ns (ns: P>0.5, **P<0.01, ***P<0.001, two-way ANOVA with Tukey post-test. The heat-shock used for experiments throughout the manuscript was 34 ⊠C for one hour. (F-L) A drop in the presynaptic SNG-1::ARGO GFP/RFP can typically be detected by 4 hours post heat-shock pulse in the presence of the Pheat-shock>Cre transgene (G-L), but not in its absence (F). Analyses are described in Materials and Methods. The data used in F are a subset of the data from E. (M) Assessing the rate of genomic recombination to remove the gfp after the pulse, applied to Day 0 adults via qPCR. Time = 0 was immediately after the 1-hour heat shock (of note, some recombination is expected to have occurred already due to the quick activation of HSF-1 to drive Phest-shock>Cre expression upon the initiation of heat-shock).

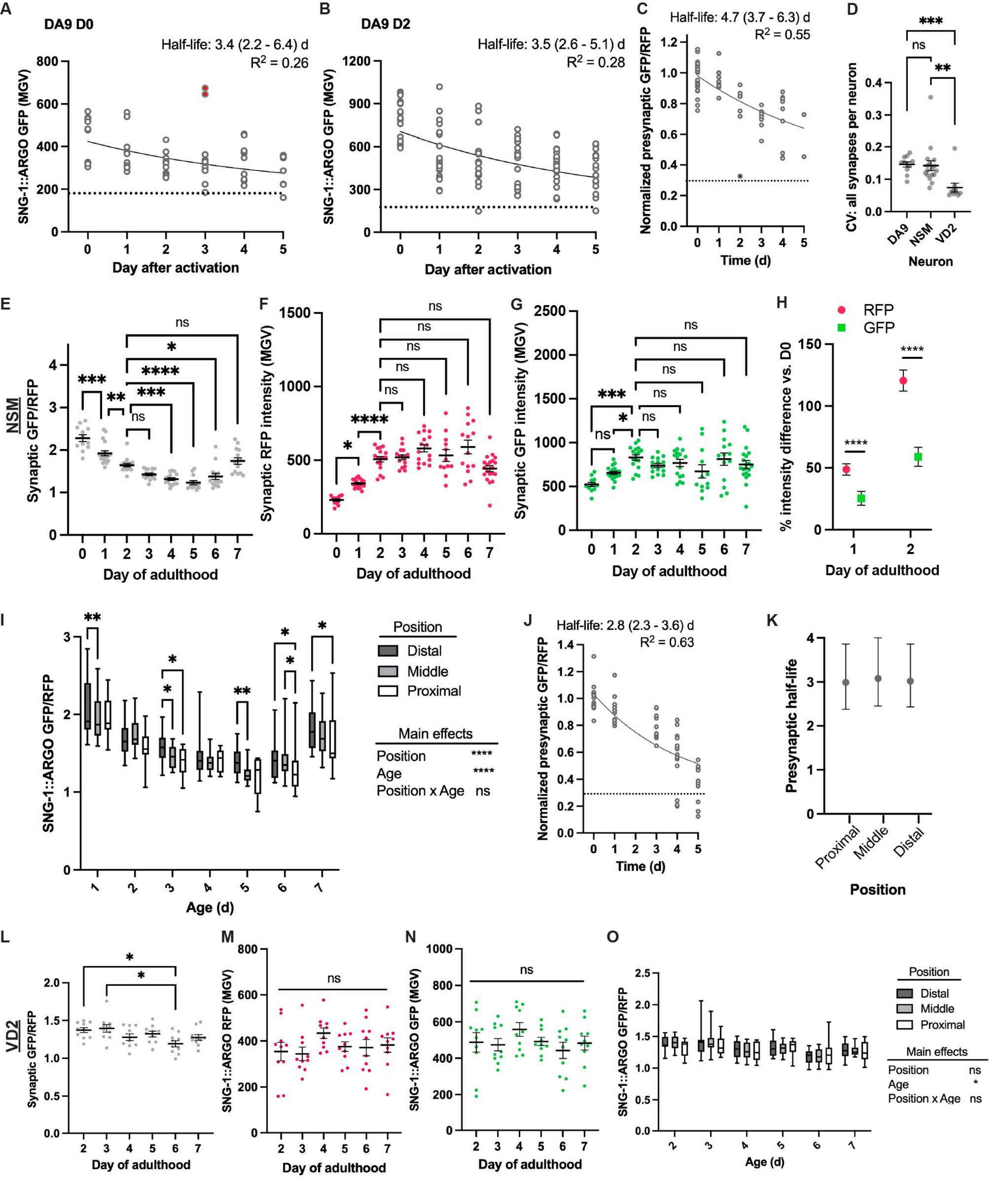

Figure S2. Additional analyses of SNG-1::ARGO steady-state fluorescence and turnover. (A-B) Quantification of SNG-1::ARGO degradation in the DA9 neuron using the mean GFP intensity at each presynapse. Each data point is the average of all the mean GFP values for one animal. Data were fit to one-phase exponential decay curves with the plateau set to the experimentally calculated plateau, which arose from autofluorescence in the GFP channel. Filled circles indicate outlier data points that were excluded from the analysis. The data for these graphs came from the same experiments as those used to generate Figures 3B and 3E. Note that for the pulse at A0, the calculated SNG-1 half-life is longer using mean GFP, which only quantifies degradation, compared to using the GFP/RFP ratio (Figure 3E), which quantifies degradation plus synapse growth. By contrast, with the pulse at A2, the half-life calculated using mean GFP is indistinguishable from that calculated using the GFP/RFP ratio, though the 95% C.I. is substantially larger. (C) A turnover experiment in the DA9 neuron with the pulse at A2, wherein the animals were maintained at 25 C after the pulse. These results are included for comparison to the uba-1(it129ts) turnover experiment; however, note that this is not the ideal comparison because the uba-1(it129ts) mutants were maintained at 25 C from the L2 larval stage. (D) Comparison of intraneuronal variance between GFP/RFP ratio across individual presynapses. Each data point is the CV for all the presynaptic puncta in one neuron. (E-G) Average presynaptic RFP/GFP ratio (E), and the underlying intensity data for RFP (F) and GFP (G) from SNG-1::ARGO in the NSM neuron. Data points are each the average of all presynaptic puncta from a single neuron; bar shows mean ± SEM. (H) Percent change in average presynaptic RFP and GFP intensity from A0 through A2. Plotted is mean ± SEM calculated from the data in F-G. (I) Steady-state SNG-1::ARGO presynaptic GFP/RFP by presynapse position along the axon in the NSM neuron. (J) One-phase exponential decay function fit to the data from a D2 SNG-1::ARGO turnover experiment in the NSM neuron; each data point shows the average presynaptic SNG-1::ARGO GFP/RFP ratio from all the presynpases in a single neuron. Dashed black line shows the experimentally calculated background, which comes from autofluorescence. Filled circles show outlier data points that were excluded from the curve calculation. (K) SNG-1::ARGO presynaptic half-life by proximal-distal position relative to the NSM neuron cell body (mean ± 95% C.I.). (L-N) Average presynaptic RFP/GFP ratio (L), and the underlying intensity data for RFP (M) and GFP (N) from SNG-1::ARGO in the VD2 neuron. Data points are each the average of all presynaptic puncta from a single neuron; bar shows mean ± SEM. (O) Steady-state SNG-1::ARGO presynaptic GFP/RFP by presynapse position along the axon in the VD2 neuron. ns: not significant, *P<0.05, **P<0.01, ***P<0.001, ****P<0.0001, one-way ANOVA or two-way ANOVA with Tukey post-test. For H and N, comparisons that were not significant are not shown.

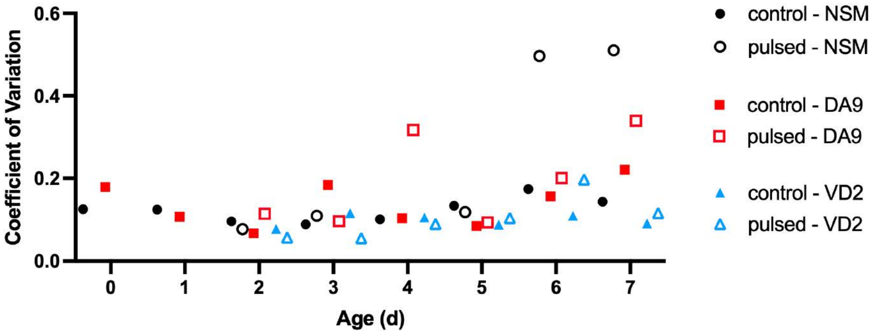

Figure S3. Interneuronal Coefficient of Variation (CV) for each neuron identity during steady-state imaging and the turnover experiment. The analyses were performed on the same datasets used throughout the manuscript for steady-state imaging and turnover with the pulse at A2. As there is one data set per neuron identity, there is one CV per neuron/age/treatment. This is therefore a qualitative assessment of SNG-1::ARGO specificity and efficacy by neuron. It complements the results in Table 1 in that it is better-designed to detect instances wherein a single allele of gfp is removed. Note that the VD2 neuron does not show higher CVs at any age compared to the DA9 and NSM neurons, so the two-phase turnover in the VD2 neuron is not due to unselective or ineffective gfp removal.

(from 1-415 bp)

cDNA sng-1 syb3140car2 (1619 bp)

CCATGCAACAACCACCATCAAACCCATAtACTCAaTCgGAAGGATATGGTTATGGAAGTTCCTATTCTCTAGAAAGTATAGGA
GGTACGTTGTTGGTGGTAGTTTGGGTATaTGAGTtAGcCTTCCTATACCAATACCTTCAAGGATAAGAGATCTTTCATATCCT

2 4 6 8 10 12 14 16 18 20 22 24 26

M Q Q P P S N P Y T Q S E G Y G Y G S S Y S L E S I G

sng-1:Argo

sng-1 C-term FRT

10 20 30 40 50 60 70 80

ACTTCAGTCTCCAAGGGAGAGGAGCTCATCAAGGAGAACATGCACATGAAGCTCTACATGGAGGGAACCGTCAACAACCACCA
TGAAGTCAGAGGTTCCCTCTCCTCGAGTAGTTCCTCTTGTACGTGTACTTCGAGATGTACCTCCCTTGGCAGTTGTTGGTGGT

28 30 32 34 36 38 40 42 44 46 48 50 52 54

T S V S K G E E L I K E N M H M K L Y M E G T V N N H H

sng-1:Argo

tagRFP

90 100 110 120 130 140 150 160

CTTCAAGTGCACCTCCGAGGGAGAGGGAAAGCCATACGAGGGAACCCAAACCATGCGTATCAAGTCGTCGAGGGAGGACCACT
GAAGTTCACGTGGAGGCTCCCTCTCCCTTTCGGTATGCTCCCTTGGGTTTGGTACGCATAGTTCAGCAGCTCCCTCCTGGTGA

56 58 60 62 64 66 68 70 72 74 76 78 80 82

F K C T S E G E G K P Y E G T Q T M R I K S S R E D H

sng-1:Argo

tagRFP

170 180 190 200 210 220 230 240

CCCATTCGCCTTCGACATCCTCGCCACCTCCTTCATGTACGGATCCCGTACCTTCATCAACCACACCCAAGGAATCCCAGACT
GGGTAAGCGGAAGCTGTAGGAGCGGTGGAGGAAGTACATGCCTAGGGCATGGAAGTAGTTGGTGTGGGTTCCTTAGGGTCTGA

84 86 88 90 92 94 96 98 100 102 104 106 108 110

S H S P S T S S P P P S C T D P V P S S T T P K E S Q T

sng-1:Argo

tagRFP

250 260 270 280 290 300 310 320 330

TCTTCAAGCAATCCTTCCCAGAGGGATTCACCTGGGAGCGTGTCACCACCTACGAGGACGGAGGAGTCCTCACCGCCACCCAA
AGAAGTTCGTTAGGAAGGGTCTCCCTAAGTGGACCCTCGCACAGTGGTGGATGCTCCTGCCTCCTCAGGAGTGGCGGTGGGTT

112 114 116 118 120 122 124 126 128 130 132 134 136 138

S S S N P S Q R D S P G S V S P P T R T E E S S P P P K

sng-1:Argo

tagRFP

340 350 360 370 380 390 400 410

cDNA sng-1 syb3140car2 (1619 bp) (from 416-830 bp)

```
GACACCTCCCTCCAAGACGGATGCCTCATCTACAACGTCAATCCGTGGAGTCAACTTCCCATCCAACGGACCAGTCATGCAAA
CTGTGGAGGGAGGTTCTGCCTACGGAGTAGATGTTGCAGTTAGGCACCTCAGTTGAAGGGTAGGTTGCCTGGTCAGTACGTTT
    140   142   144   146   148   150   152   154   156   158   160   162   164
  T  P  P  S  K  T  D  A  S  S  T  T  S  I  R  G  V  N  F  P  S  N  G  P  V  M  Q
sng-1:Argo
tagRFP
  420       430       440       450       460       470       480       490
```

```
AGAAGACCCTCGGATGGGAGGCCAACACCGAGATGCTCTACCCAGCCGACGGAGGACTcGAGGGACGTACCGACATGGCCCTC
TCTTCTGGGAGCCTACCCTCCGGTTGTGGCTCTACGAGATGGGTCGGCTGCCTCCTGAgCTCCCTGCATGGCTGTACCGGGAG
166   168   170   172   174   176   178   180   182   184   186   188   190   192
 K K  T  L  G  W  E  A  N  T  E  M  L  Y  P  A  D  G  G  L  E  G  R  T  D  M  A  L
sng-1:Argo
tagRFP
500       510       520       530       540       550       560       570       580
```

```
AAGCTCGTCGGAGGAGGACACCTCATCTGCAACTTCAAGACCACCTACCGTTCCAAGCCAGCCAAGAACCTCAAGATGCCAGG
TTCGAGCAGCCTCCTCCTGTGGAGTAGACGTTGAAGTTCTGGTGGATGGCAAGGTTCGGTCGGTTCTTGGAGTTCTACGGTCC
194   196   198   200   202   204   206   208   210   212   214   216   218   220
 K  L  V  G  G  G  H  L  I  C  N  F  K  T  T  Y  R  S  K  P  A  K  N  L  K  M  P  G
sng-1:Argo
tagRFP
       590       600       610       620       630       640       650       660
```

```
AGTCTACTACGTCGACCACCGTCTcGAGCGTATCAAGGAGGCCGACAAGGAGACcTACGTCGAGCAACACGAGGTCGCCGTCG
TCAGATGATGCAGCTGGTGGCAGAgCTCGCATAGTTCCTCCGGCTGTTCCTCTGgATGCAGCTCGTTGTGCTCCAGCGGCAGC
  222   224   226   228   230   232   234   236   238   240   242   244   246   248
  V  Y  Y  V  D  H  R  L  E  R  I  K  E  A  D  K  E  T  Y  V  E  Q  H  E  V  A  V
sng-1:Argo
tagRFP
     670       680       690       700       710       720       730       740
```

```
CCCGTTACTGCGACCTCCCATCCAAGCTCGGACACAAGCTCAACGgTatggaTgaActGtaTaaGGGTTCTGGGAGTGGAAGC
GGGCAATGACGCTGGAGGGTAGGTTCGAGCCTGTGTTCGAGTTGCcAtacctActTgaCatAttCCCAAGACCCTCACCTTCG
  250   252   254   256   258   260   262   264   266   268   270   272   274   276
 A  R  Y  C  D  L  P  S  K  L  G  H  K  L  N  G  M  D  E  L  Y  K  G  S  G  S  G  S
sng-1:Argo
tagRFP                                                        GSGSGSGS linker
  750       760       770       780       790       800       810       820       830
```

cDNA sng-1 syb3140car2 (1619 bp) (from 831-1245 bp)

GGCTCTATGAGTAAAGGAGAAGAACTTTTCACTGGAGTTGTCCCAATTCTTGTTGAATTAGATGGTGATGTTAATGGGCACAA
CCGAGATACTCATTTCCTCTTCTTGAAAAGTGACCTCAACAGGGTTAAGAACAACTTAATCTACCACTACAATTACCCGTGTT

278 280 282 284 286 288 290 292 294 296 298 300 302 304

G S M S K G E E L F T G V V P I L V E L D G D V N G H K

sng-1:Argo

GFP

840 850 860 870 880 890 900 910

ATTTTCTGTCAGTGGAGAGGGTGAAGGTGATGCAACATACGGAAAACTTACCCTTAAATTTATTTGCACTACTGGAAAACTAC
TAAAAGACAGTCACCTCTCCCACTTCCACTACGTTGTATGCCTTTTGAATGGGAATTTAAATAAACGTGATGACCTTTTGATG

306 308 310 312 314 316 318 320 322 324 326 328 330

F S V S G E G E G D A T Y G K L T L K F I C T T G K L

sng-1:Argo

GFP

920 930 940 950 960 970 980 990

CTGTTCCATGGCCAACACTTGTCACTACTTTCTGTTATGGTGTTCAATGCTTCTCGAGATACCCAGATCATATGAAACGGCAT
GACAAGGTACCGGTTGTGAACAGTGATGAAAGACAATACCACAAGTTACGAAGAGCTCTATGGGTCTAGTATACTTTGCCGTA

332 334 336 338 340 342 344 346 348 350 352 354 356 358

P V P W P T L V T T F C Y G V Q C F S R Y P D H M K R H

sng-1:Argo

GFP

1,000 1,010 1,020 1,030 1,040 1,050 1,060 1,070

GACTTTTTCAAGAGTGCCATGCCCGAAGGTTATGTACAGGAAAGAACTATATTTTTCAAAGATGACGGGAACTACAAGACACG
CTGAAAAAGTTCTCACGGTACGGGCTTCCAATACATGTCCTTTCTTGATATAAAAAGTTTCTACTGCCCTTGATGTTCTGTGC

360 362 364 366 368 370 372 374 376 378 380 382 384 386

D F F K S A M P E G Y V Q E R T I F F K D D G N Y K T R

sng-1:Argo

GFP

1,080 1,090 1,100 1,110 1,120 1,130 1,140 1,150 1,160

TGCTGAAGTCAAGTTTGAAGGTGATACCCTTGTTAATAGAATCGAGTTAAAAGGTATTGATTTTAAAGAAGATGGAAACATTC
ACGACTTCAGTTCAAACTTCCACTATGGGAACAATTATCTTAGCTCAATTTTCCATAACTAAAATTTCTTCTACCTTTGTAAG

388 390 392 394 396 398 400 402 404 406 408 410 412 414

A E V K F E G D T L V N R I E L K G I D F K E D G N I

sng-1:Argo

GFP

1,170 1,180 1,190 1,200 1,210 1,220 1,230 1,240

cDNA sng-1 syb3140car2 (1619 bp) (from 1246-1619 bp)

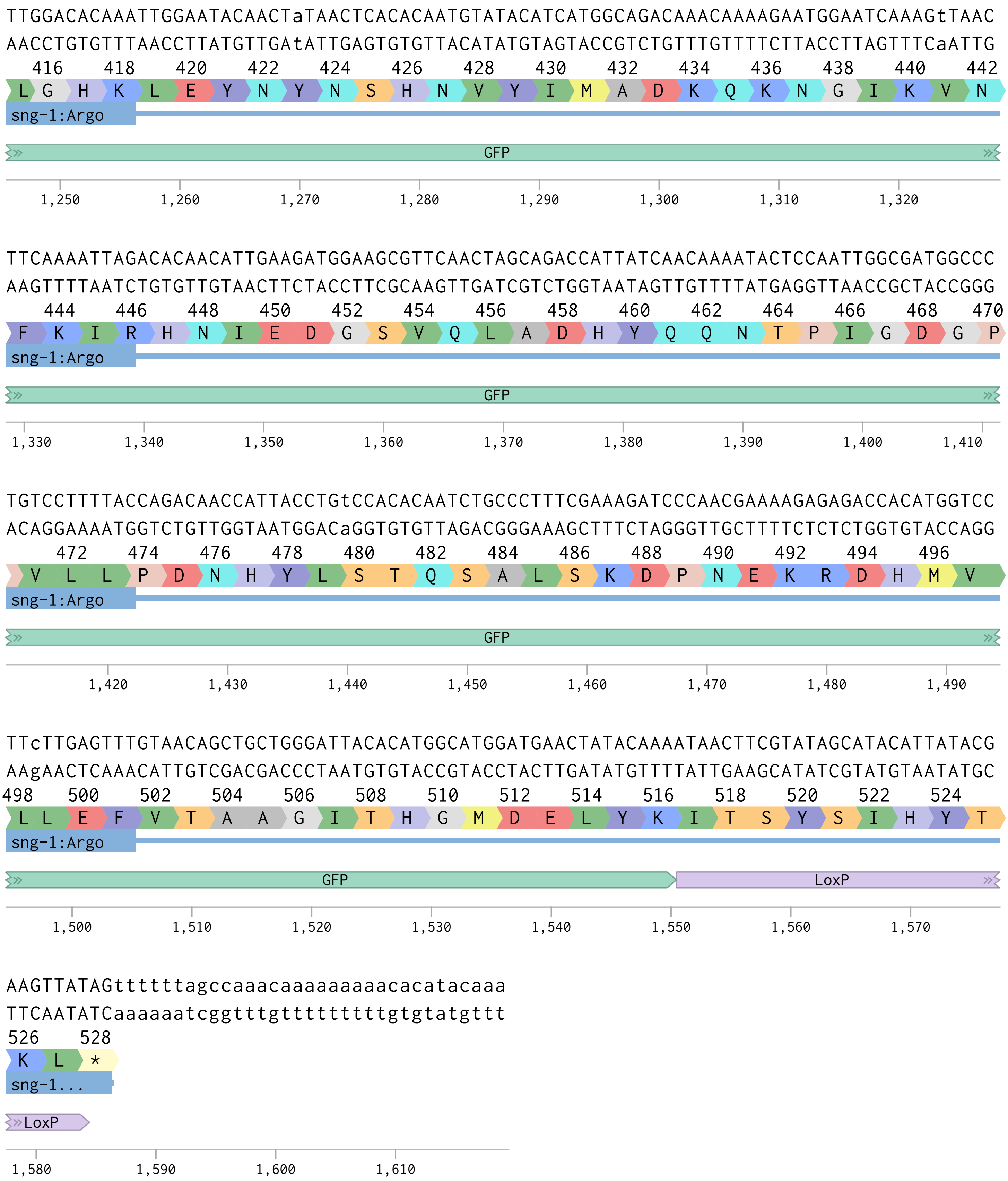

Figure S4. Sequence of sng-1(syb1340car2) cDNA when dually-tagged with RFP and GFP.

(from 1-465 bp)

syb3140car2 recombined (891 bp)

```
CCATGCAACAACCACCATCAAACCCATAtACTCAaTCgGAAGGATATGGTTATGGAAGTTCCTATTCTCTAGAAAGTATAGGAACTTCAGTCT
GGTACGTTGTTGGTGGTAGTTTGGGTATaTGAGTtAGcCTTCCTATACCAATACCTTCAAGGATAAGAGATCTTTCATATCCTTGAAGTCAGA
     2   4   6   8   10  12  14  16  18  20  22  24  26  28  30
   M Q Q P P S N P Y T Q S E G Y G Y G S S Y S L E S I G T S V
Translation
sng-1 C-term    FRT    
10 20 30 40 50 60 70 80 90
```

```
CCAAGGGAGAGGAGCTCATCAAGGAGAACATGCACATGAAGCTCTACATGGAGGGAACCGTCAACAACCACCACTTCAAGTGCACCTCCGAGG
GGTTCCCTCTCCTCGAGTAGTTCCTCTTGTACGTGTACTTCGAGATGTACCTCCCTTGGCAGTTGTTGGTGGTGAAGTTCACGTGGAGGCTCC
  32  34  36  38  40  42  44  46  48  50  52  54  56  58  60
 S K G E E L I K E N M H M K L Y M E G T V N N H H F K C T S E
Translation
tagRFP
100 110 120 130 140 150 160 170 180
```

```
GAGAGGGAAAGCCATACGAGGGAACCCAAACCATGCGTATCAAGGTCGTCGAGGGAGGACCACTCCCATTCGCCTTCGACATCCTCGCCACCT
CTCTCCCTTTCGGTATGCTCCCTTGGGTTTGGTACGCATAGTTCCAGCAGCTCCCTCCTGGTGAGGGTAAGCGGAAGCTGTAGGAGCGGTGGA
62  64  66  68  70  72  74  76  78  80  82  84  86  88  90  92
 G E G K P Y E G T Q T M R I K V V E G G P L P F A F D I L A T
Translation
tagRFP
190 200 210 220 230 240 250 260 270
```

```
CCTTCATGTACGGATCCCGTACCTTCATCAACCACACCCAAGGAATCCCAGACTTCTTCAAGCAATCCTTCCCAGAGGGATTCACCTGGGAGC
GGAAGTACATGCCTAGGGCATGGAAGTAGTTGGTGTGGGTTCCTTAGGGTCTGAAGAAGTTCGTTAGGAAGGGTCTCCCTAAGTGGACCCTCG
  94  96  98  100 102 104 106 108 110 112 114 116 118 120 122
 S F M Y G S R T F I N H T Q G I P D F F K Q S F P E G F T W E
Translation
tagRFP
280 290 300 310 320 330 340 350 360 370
```

```
GTGTCACCACCTACGAGGACGGAGGAGTCCTCACCGCCACCCAAGACACCTCCCTCCAAGACGGATGCCTCATCTACAACGTCAAGATCCGTG
CACAGTGGTGGATGCTCCTGCCTCCTCAGGAGTGGCGGTGGGTTCTGTGGAGGGAGGTTCTGCCTACGGAGTAGATGTTGCAGTTCTAGGCAC
124 126 128 130 132 134 136 138 140 142 144 146 148 150 152 154
 R V T T Y E D G G V L T A T Q D T S L Q D G C L I Y N V K I R
Translation
tagRFP
380 390 400 410 420 430 440 450 460
```

syb3140car2 recombined (891 bp) (from 466-891 bp)

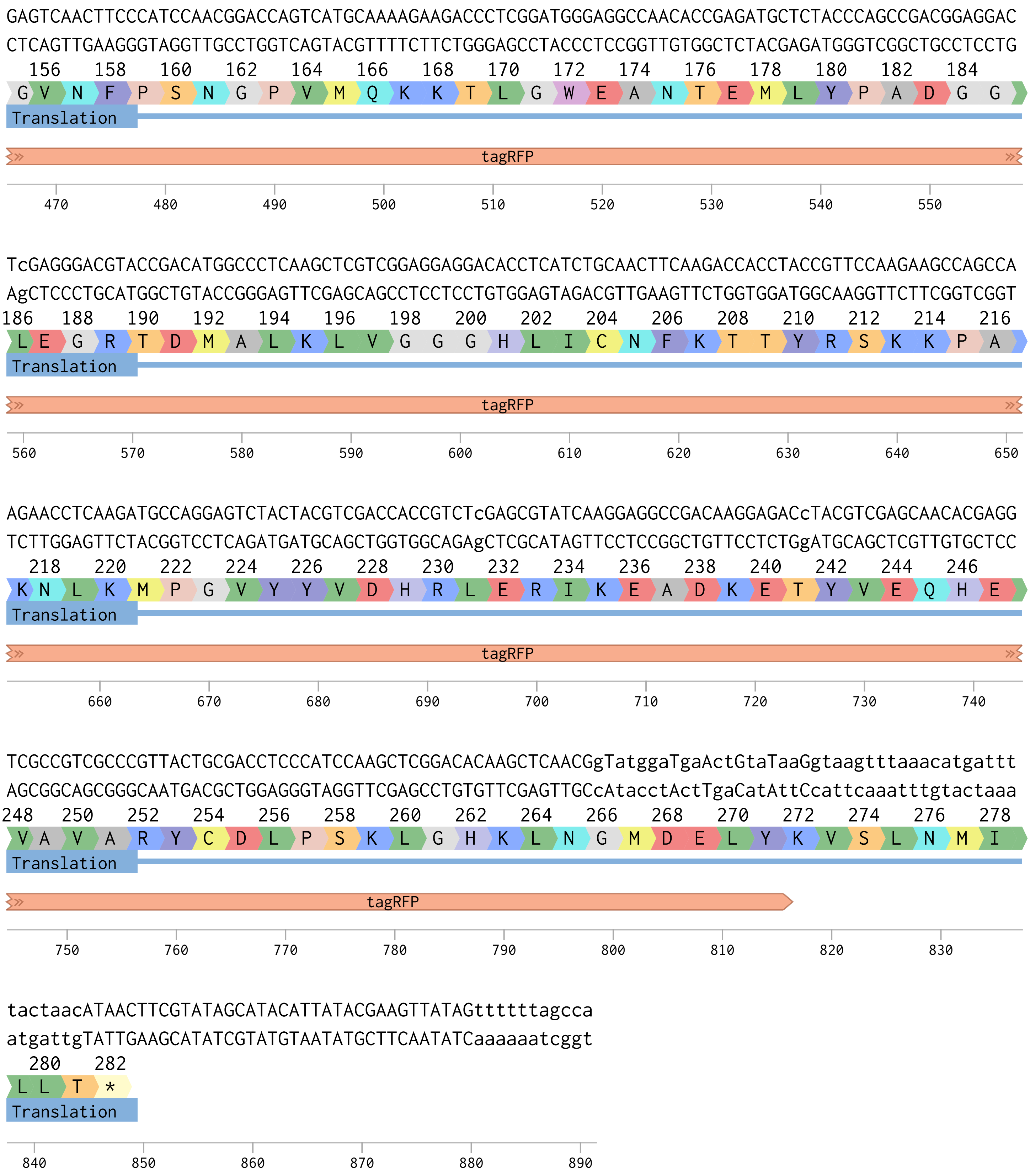

Figure S5. Sequence of sng-1(syb3140car2) cDNA after Cre/LoxP-mediated removal of GFP.

(from 1-749 bp)

sng-1 syb3140car2 genomic locus (2457 bp)

```
GCAACAACCACCATCAAACCCATAtACTCAaTCgGAAGGATATGGTTATGGAAGTTCCTATTCTCTAGAAAGTATAGGAACTTCATAAatttcaaattttaaatac
CGTTGTTGGTGGTAGTTTGGGTATaTGAGTtAGcCTTCCTATACCAATACCTTCAAGGATAAGAGATCTTTCATATCCTTGAAGTATTtaaagtttaaaatttatg
```

FRT 3' utr

10 20 30 40 50 60 70 80 90 100

```
tgaatatttgtttttttcctattatttatttattctctttgtgtttttttcttgctttctaaaaaattaattcaatccaaatctaaacatttttttttctctttc
acttataaacaaaaaaaggataataaataaataagagaaacacaaaaaaagaacgaaagattttttaattaagttaggtttagatttgtaaaaaaaaagagaaag
```

3' utr

110 120 130 140 150 160 170 180 190 200 210

```
cgtctcccaattcgtattccgctcctctcatctgaacacaatgtgcaagtttatttatcttctcgctttcatttcattaggacgtgggggaattggtggaagggggg
gcagagggttaagcataaggcgaggagagtagacttgtgttacacgttcaaataaatagaagagcgaaagtaaagtaatcctgcaccccccttaaccaccttccccc
```

3' utr

220 230 240 250 260 270 280 290 300 310 320

```
aaacacacaaaaggatgatggaaatgaaataaggacacacaatatgcaacaacattcaattcagaaatatggaggaaggtttaaaagaaaacataaaaatatataga
tttgtgtgttttcctactacctttactttattcctgtgtgttatacgttgttgtaagttaagtctttatacctccttccaaattttcttttgtatttttatatatct
```

3' utr

330 340 350 360 370 380 390 400 410 420

```
ggaggaaggaaaactagtaaaaaataagcaaagaaattaggcgaacgatgagaattgtcctcgcttgGAAGTTCCTATTCTCTAGAAAGTATAGGAACTTCAGTCTC
cctccttccttttgatcatttttattcgtttctttaatccgcttgctactcttaacaggagcgaacCTTCAAGGATAAGAGATCTTTCATATCCTTGAAGTCAGAG
```

1 2
V S

3' utr FRT

430 440 450 460 470 480 490 500 510 520 530

```
CAAGGGAGAGGAGCTCATCAAGGAGAACATGCACATGAAGCTCTACATGGAGGGAACCGTCAACAACCACCACTTCAAGTGCACCTCCGAGGGAGAGGGAAAGCCAT
GTTCCCTCTCCTCGAGTAGTTCCTCTTGTACGTGTACTTCGAGATGTACCTCCCTTGGCAGTTGTTGGTGGTGAAGTTCACGTGGAGGCTCCCTCTCCCTTTCGGTA
```

3 4 5 6 7 8 9 10 11 12 13 14 15 16 17 18 19 20 21 22 23 24 25 26 27 28 29 30 31 32 33 34 35 36 37
K G E E L I K E N M H M K L Y M E G T V N N H H F K C T S E G E G K P

tagRFP

540 550 560 570 580 590 600 610 620 630 640

```
ACGAGGGAACCCAAACCATGCGTATCAAGGtaagtttaaacatatatatactaactaaccctgattatttaaattttcaggTCGTCGAGGGAGGACCACTCCCATTC
TGCTCCCTTGGGTTTGGTACGCATAGTTCCattcaaatttgtatatatatgattgattgggactaataaatttaaaagtccAGCAGCTCCCTCCTGGTGAGGGTAAG
```

38 39 40 41 42 43 44 45 46 47
Y E G T Q T M R I K

tagRFP

1 2 3 4 5 6 7 8 9
S S R E D H S H S

tagRFP

intron

650 660 670 680 690 700 710 720 730 740

sng-1 syb3140car2 genomic locus (2457 bp) (from 750-1391 bp)

GCCTTCGACATCCTCGCCACCTCCTTCATGTACGGATCCCGTACCTTCATCAACCACACCCAAGGAATCCCAGACTTCTTCAAGCAATCCTTCCCAGAGGGATTCAC
CGGAAGCTGTAGGAGCGGTGGAGGAAGTACATGCCTAGGGCATGGAAGTAGTTGGTGTGGGTTCCTTAGGGTCTGAAGAAGTTCGTTAGGAAGGGTCTCCCTAAGTG
10 11 12 13 14 15 16 17 18 19 20 21 22 23 24 25 26 27 28 29 30 31 32 33 34 35 36 37 38 39 40 41 42 43 44
P S T S S P P P S C T D P V P S S T T P K E S Q T S S S N P S Q R D S
tagRFP
750 760 770 780 790 800 810 820 830 840 850

CTGGGAGCGTGTCACCACCTACGAGGACGGAGGAGTCCTCACCGCCACCCAAGACACCTCCCTCCAAGACGGATGCCTCATCTACAACGTCAAGgtaagtttaaaca
GACCCTCGCACAGTGGTGGATGCTCCTGCCTCCTCAGGAGTGGCGGTGGGTTCTGTGGAGGGAGGTTCTGCCTACGGAGTAGATGTTGCAGTTCcattcaaatttgt
45 46 47 48 49 50 51 52 53 54 55 56 57 58 59 60 61 62 63 64 65 66 67 68 69 70 71 72 73 74 75
P G S V S P P T R T E E S S P P P K T P P S K T D A S S T T S
tagRFP
intron
860 870 880 890 900 910 920 930 940 950 960

gttcggtactaactaaccatacatatttaaattttcagATCCGTGGAGTCAACTTCCCATCCAACGGACCAGTCATGCAAAAGAAGACCCTCGGATGGGAGGCCAAC
caagccatgattgattggtatgtataaatttaaaagtcTAGGCACCTCAGTTGAAGGGTAGGTTGCCTGGTCAGTACGTTTTCTTCTGGGAGCCTACCCTCCGGTTG
1 2 3 4 5 6 7 8 9 10 11 12 13 14 15 16 17 18 19 20 21 22 23
I R G V N F P S N G P V M Q K K T L G W E A N
tagRFP
intron
970 980 990 1,000 1,010 1,020 1,030 1,040 1,050 1,060 1,070

ACCGAGATGCTCTACCCAGCCGACGGAGGACTcGAGGGACGTACCGACATGGCCCTCAAGCTCGTCGGAGGAGGACACCTCATCTGCAACTTCAAGACCACCTACCG
TGGCTCTACGAGATGGGTCGGCTGCCTCCTGAgCTCCCTGCATGGCTGTACCGGGAGTTCGAGCAGCCTCCTCCTGTGGAGTAGACGTTGAAGTTCTGGTGGATGGC
24 25 26 27 28 29 30 31 32 33 34 35 36 37 38 39 40 41 42 43 44 45 46 47 48 49 50 51 52 53 54 55 56 57 58 59
T E M L Y P A D G G L E G R T D M A L K L V G G G H L I C N F K T T Y R
tagRFP
1,080 1,090 1,100 1,110 1,120 1,130 1,140 1,150 1,160 1,170

TTCCAAGgtaagtttaaacatgattttactaactaactaatctgatttaaattttcagAAGCCAGCCAAGAACCTCAAGATGCCAGGAGTCTACTACGTCGACCACC
AAGGTTCcattcaaatttgtactaaaatgattgattgattagactaaatttaaaagtcTTCGGTCGGTTCTTGGAGTTCTACGGTCCTCAGATGATGCAGCTGGTGG
60
S
1 2 3 4 5 6 7 8 9 10 11 12 13 14 15 16
K P A K N L K M P G V Y Y V D H
tagRFP
intron
1,180 1,190 1,200 1,210 1,220 1,230 1,240 1,250 1,260 1,270 1,280

GTCTcGAGCGTATCAAGGAGGCCGACAAGGAGACcTACGTCGAGCAACACGAGGTCGCCGTCGCCCGTTACTGCGACCTCCCATCCAAGCTCGGACACAAGCTCAAC
CAGAgCTCGCATAGTTCCTCCGGCTGTTCCTCTGgATGCAGCTCGTTGTGCTCCAGCGGCAGCGGGCAATGACGCTGGAGGGTAGGTTCGAGCCTGTGTTCGAGTTG
17 18 19 20 21 22 23 24 25 26 27 28 29 30 31 32 33 34 35 36 37 38 39 40 41 42 43 44 45 46 47 48 49 50 51 52
R L E R I K E A D K E T Y V E Q H E V A V A R Y C D L P S K L G H K L N
tagRFP
1,290 1,300 1,310 1,320 1,330 1,340 1,350 1,360 1,370 1,380 1,390

sng-1 syb3140car2 genomic locus (2457 bp) (from 1392-2033 bp)

GgTatggaTgaActGtaTaaGgtaagtttaaacatgattttactaacATAACTTCGTATAGCATACATTATACGAAGTTATtaactaatctgatttaaattttcagG
CcAtacctActTgaCatAttCcattcaaatttgtactaaaatgattgTATTGAAGCATATCGTATGTAATATGCTTCAATAattgattagactaaatttaaaagtcC
53 54 55 56 57 58 59
G M D E L Y K
tagRFP
intron loxP intron
1,400 1,410 1,420 1,430 1,440 1,450 1,460 1,470 1,480 1,490

GTTCTGGGAGTGGAAGCGGCTCTATGAGTAAAGGAGAAGAACTTTTCACTGGAGTTGTCCCAATTCTTGTTGAATTAGATGGTGATGTTAATGGGCACAAATTTTCT
CAAGACCCTCACCTTCGCCGAGATACTCATTTCCTCTTCTTGAAAAGTGACCTCAACAGGGTTAAGAACAACTTAATCTACCACTACAATTACCCGTGTTTAAAAGA
1 2 3 4 5 6 7 8 9 10 11 12 13 14 15 16 17 18 19 20 21 22 23 24 25 26 27 28
M S K G E E L F T G V V P I L V E L D G D V N G H K F S
GFP
GSGSGSGS linker
1,500 1,510 1,520 1,530 1,540 1,550 1,560 1,570 1,580 1,590 1,600

GTCAGTGGAGAGGGTGAAGGTGATGCAACATACGGAAAACTTACCCTTAAATTTATTTGCACTACTGGAAAACTACCTGTTCCATGGGTAAGTTTAAACATATATAT
CAGTCACCTCTCCCACTTCCACTACGTTGTATGCCTTTTGAATGGGAATTTAAATAAACGTGATGACCTTTTGATGGACAAGGTACCCATTCAAATTTGTATATATA
29 30 31 32 33 34 35 36 37 38 39 40 41 42 43 44 45 46 47 48 49 50 51 52 53 54 55 56 57
V S G E G E G D A T Y G K L T L K F I C T T G K L P V P W
GFP
intron
1,610 1,620 1,630 1,640 1,650 1,660 1,670 1,680 1,690 1,700 1,710

ACTAACTAACCCTGATTATTTAAATTTTCAGCCAACACTTGTCACTACTTTCTGTTATGGTGTTCAATGCTTCTCGAGATACCCAGATCATATGAAACGGCATGACT
TGATTGATTGGGACTAATAAATTTAAAAGTCGGTTGTGAACAGTGATGAAAGACAATACCACAAGTTACGAAGAGCTCTATGGGTCTAGTATACTTTGCCGTACTGA
1 2 3 4 5 6 7 8 9 10 11 12 13 14 15 16 17 18 19 20 21 22 23 24 25
P T L V T T F C Y G V Q C F S R Y P D H M K R H D
GFP
intron
1,720 1,730 1,740 1,750 1,760 1,770 1,780 1,790 1,800 1,810

TTTTCAAGAGTGCCATGCCCGAAGGTTATGTACAGGAAAGAACTATATTTTTCAAAGATGACGGGAACTACAAGACACGTAAGTTTAAACAGTTCGGTACTAACTAA
AAAAGTTCTCACGGTACGGGCTTCCAATACATGTCCTTTCTTGATATAAAAAGTTTCTACTGCCCTTGATGTTCTGTGCATTCAAATTTGTCAAGCCATGATTGATT
26 27 28 29 30 31 32 33 34 35 36 37 38 39 40 41 42 43 44 45 46 47 48 49 50 51
F F K S A M P E G Y V Q E R T I F F K D D G N Y K T
GFP
intron
1,820 1,830 1,840 1,850 1,860 1,870 1,880 1,890 1,900 1,910 1,920

CCATACATATTTAAATTTTCAGGTGCTGAAGTCAAGTTTGAAGGTGATACCCTTGTTAATAGAATCGAGTTAAAAGGTATTGATTTTAAAGAAGATGGAAACATTCT
GGTATGTATAAATTTAAAAGTCCACGACTTCAGTTCAAACTTCCACTATGGGAACAATTATCTTAGCTCAATTTTCCATAACTAAAATTTCTTCTACCTTTGTAAGA
1 2 3 4 5 6 7 8 9 10 11 12 13 14 15 16 17 18 19 20 21 22 23 24 25 26 27 28
A E V K F E G D T L V N R I E L K G I D F K E D G N I L
GFP
intron
1,930 1,940 1,950 1,960 1,970 1,980 1,990 2,000 2,010 2,020 2,030

sng-1 syb3140car2 genomic locus (2457 bp) (from 2034-2457 bp)

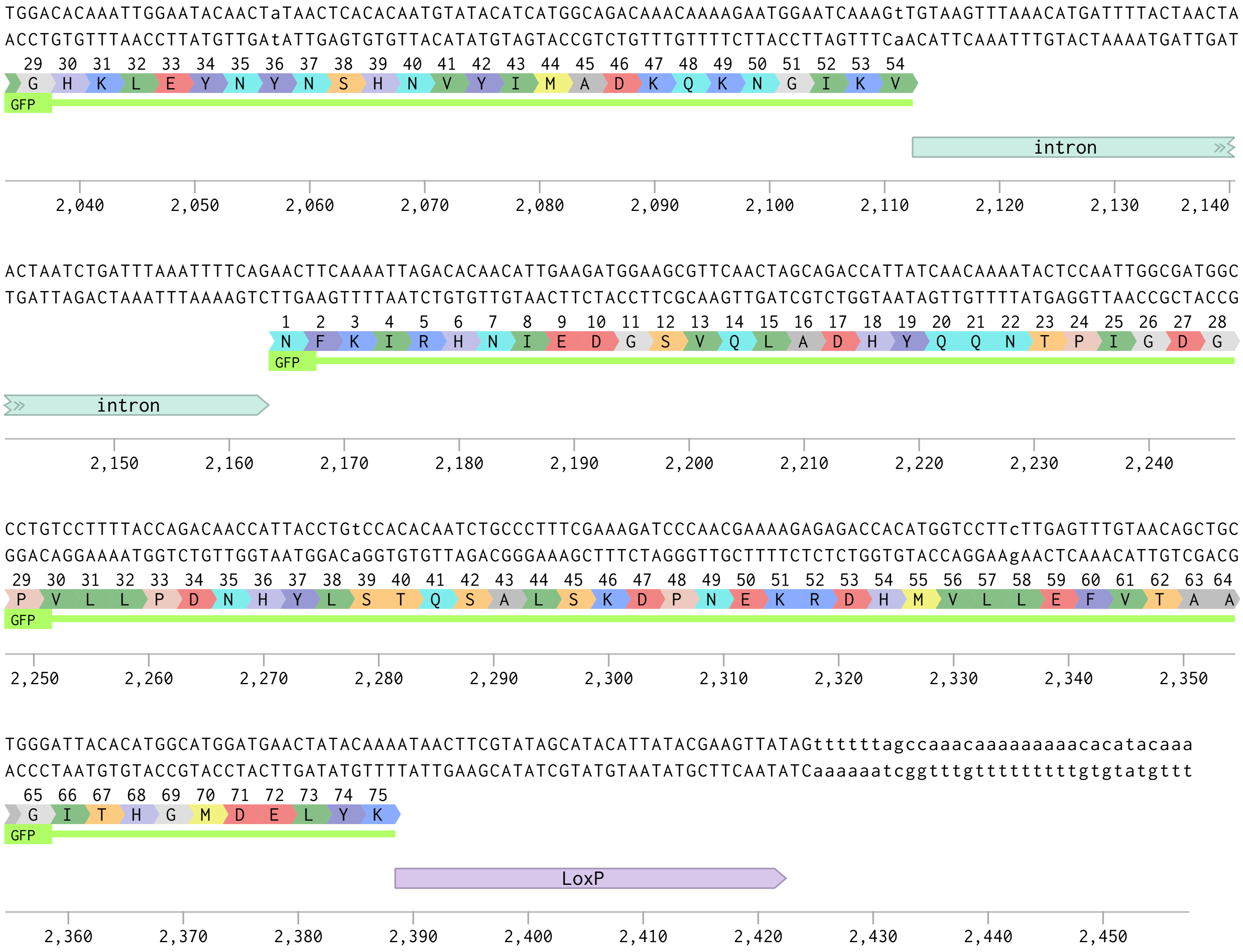

Fig S6. Annotated genomic sequence of sng-1(syb3140car2[argo]).
